# A national analysis of the pharmacy workforce in Indonesia

**DOI:** 10.1186/s12960-022-00767-4

**Published:** 2022-09-29

**Authors:** Sherly Meilianti, Felicity Smith, Franciscus Kristianto, Roy Himawan, Desak Ketut Ernawati, Rasta Naya, Ian Bates

**Affiliations:** 1 Indonesian Pharmacists Association, Wijaya Kusuma No. 17, Jatipulo, West Jakarta, Jakarta 11430 Indonesia; 2 Department of Practice and Policy, School of Pharmacy, University College London, 29-39 Brunswick Square, Bloomsbury, London, WC1N 1AX United Kingdom; 3 International Pharmaceutical Federation, Andries Bickerweg 5, 2517 JP The Hague, Netherlands; 4 Faculty of Pharmacy, University of Surabaya, Jalan Raya Kalirungkut, Surabaya, 60293 Indonesia; 5 Pharmaceutical and Medical Devices, Ministry of Health, Jakarta, 12950 Indonesia; 6 Department of Pharmacology and Therapy, Universitas Udayana, Denpasar, Bali 80234 Indonesia

**Keywords:** Capacity, Indonesia, Pharmacist, Policy

## Abstract

**Background:**

Pharmacists play a fundamental role in healthcare systems and achieving Universal Health Coverage (UHC) through quality primary healthcare service provision. While the World Health Organization (WHO) forecasts a global shortage of health workforce by 2030, mainly affecting low- and middle-income nations (LMICs), limited published literature is found regarding pharmacy workforce capacity in LMICs, including Indonesia. This paper aims to analyse pharmacists’ capacity in Indonesia to identify emerging workforce planning gaps for future workforce planning and policies in Indonesia.

**Method:**

Several data sources were accessed, including a database from the National Pharmacy Committee and the professional leadership body in Indonesia. Descriptive (frequencies, percentages, and mean), correlational and time-series analysis using curve estimation were conducted. Secondary data on the number of programmes, pharmacy students, pharmacy workforce (pharmacists and pharmacy technicians) per province were obtained from the Ministry websites and reports.

**Result:**

There were a total of 77 191 registered pharmacists in Indonesia in 2019. The pharmacists’ pyramid showed a youth bulge as a general indication of market expansion in the education sector correlating to the pharmacy programme’s number and size. There was a variation in pharmacy workforce density and access to pharmacy programmes across islands, which also were strongly correlated. Forecasting estimates that by 2030, women will represent around 86% of pharmacists in Indonesia. More female pharmacists were found working in the hospital and primary healthcare (providing direct services to patients) than male pharmacists. Younger pharmacists worked in the industrial sector, while older pharmacists worked in governmental and educational institutions.

**Conclusion:**

This study signposted workforce planning gaps for policy development in Indonesia, including a need to develop structured training to support early career pharmacists in their practice. There is also a need for better access to professional development programmes designed to support female pharmacists return to the regulated workforce following career breaks. National policy to promote equitable distribution and retention of pharmacists is recommended.

## Background

Investment in the health workforce is a critical point in achieving the Universal Health Coverage and Sustainable Development Goals. The World Health Organization (WHO) and Global Health Workforce Alliance are clear that there can be no health care delivery without a capable healthcare workforce [1]. In 2021, the WHO established ten global health issues to track, including “advance health for all” through strengthening the global health workforce [2]. The WHO launched a global campaign in 2021 to highlight an urgent need to invest in health and care workers and designated 2021 as the International Year of Health and Care Workers [3]. Pharmacists, one of the health and care workers, have essential roles in delivering direct healthcare services, and they are known as the most accessible and approachable health workforces in communities in some countries [4]. The COVID-19 pandemic has proven that pharmacists have diverse roles during the pandemic, such as public health, including disease prevention and infection control, information dissemination, and medication management, including medicines supply, patient care and support for healthcare professionals [5, 6]. Hence, the rise of demands for the quality pharmacy workforce is unneglectable. The role of the pharmacy workforce, particularly in primary health services, has never been stronger, especially after the commitment in Kazakhstan, Astana Declaration on Primary Health Care [4, 7]. The pharmacy workforce plays a fundamental role in healthcare systems and achieving Universal Health Coverage through quality primary healthcare service provision [4]. Health workforce investment is not only related to the number, but also related to tailored policies reflecting on the workforce characteristic and behaviours. Evidence-based analysis and data to inform workforce planning for policy-making at the national level are imperative [8]. This is also aligned with one of the development goals (DGs) developed by the International Pharmaceutical Federation (FIP), DG number 12: Pharmacy intelligence [9]. The workforce element of this DG is related to the availability of national strategy and action on collecting and sharing workforce data and workforce planning for workforce development [9].

There was limited published literature found regarding pharmacy workforce development in developing countries [10]. A global systematic review on the pharmacy workforce highlighted that increases in recruitment and retention, as well as reductions in attrition, will be necessary to maintain and expand the future pharmacy workforce [10]. A global trends publication on the pharmacy workforce shows that low and lower-middle-income countries tend to have a lower workforce density of pharmacists compared to high and upper-middle-income countries [11]. A global report analysing pharmacy workforce trends over 2006–2016 showed a widening income-based “capacity gap” between countries and that this gap will continue to widen [12]. Although there are no global benchmarks for assessing the sufficiency of the workforce, a relative lower density suggests an inadequate capacity to meet minimum coverage of essential services [13]. Further research on the supply-side influences and a needs-based understanding of shortages is required [12]. In Indonesia itself, a national needs assessment of the pharmacy workforce highlighted a need to develop a national project on pharmacy workforce intelligence [14]. This paper aims to analyse pharmacists’ capacity in Indonesia, add future workforce predictions and identify emerging workforce planning gaps. Identifying gaps and discussing the approach to improving datasets could be the basis for future workforce planning and policies in Indonesia.

### Country context

As the fourth most populous country in the world, Indonesia has more than 267 million population [15]. The population pyramid revealed a late growing stage. Death and birth rates are dropping at a slower rate, and population growth has become steadier [16]. The demographic changes show an increase in the ageing population with a smaller fraction of the reproductive-age population. This demographic shift influences an epidemiologic transition in which non-communicable diseases (NCDs) are increasingly important while infectious diseases remain a significant part of the disease burden [17]. As a result of this transition, the population require human resource for health (HRH) with varied specialisms, skilled in the use of technology, and a high level of elderly care. An ageing population also bring an ageing workforce which affects the future supply of HRH and their capacity to perform physically demanding work [8].

Geographically, Indonesia is recognised as the largest archipelago globally, consisting of more than 17 000 islands. This unique demographic may affect the access to healthcare facilities in the country. Administratively, the territory of Indonesia is divided into 34 provinces. For comparison purposes, in this paper, the territories were divided into six categories of islands: (1) Sumatera, (2) Java, (3) Bali and Lombok, (4) Kalimantan, (5) Sulawesi, and (6) Maluku and Papua islands. Fifty-six per cent of the population lives on Java island, which is the world’s most populous island [18]. This is followed by Sumatera (21.68%), Sulawesi (7.36%), Kalimantan (6.15%), Bali and Nusa Tenggara (5.54%) and Maluku and Papua (3.17%) [19]. As part of the subpopulation of the general population, these imbalances may also bring challenges to the health workforce.

Indonesia faces several opportunities and challenges with regard to ensuring adequate numbers, geographic distribution, and a mix of specialisations of the HRH to provide healthcare services [20]. Consequently, HRH development is one focus of the Ministry of Health strategic plan 2020–2024 to strengthen the health system [21]. This includes strategic activities related to “HRH planning and management, pre-service and in-service training, HRH quality including registration and certification and other management and technical support for HRH development programme” [21]. Some strategies have been initiated to tackle general HRH challenges in Indonesia, including (1) increasing HRH production by opening public universities and partnerships with private universities; (2) recruiting HRH through many different types of contracts, including special assignments to work in rural areas and providing support through scholarship or additional incentives to HRH in rural areas; (3) accreditation of training institutions; (4) strengthening health workforce information systems and building a data-driven culture to address HRH challenges [22–25].

Together with physicians and nursing-midwifery practitioners, the pharmacy workforce in Indonesia makes up 85% of the total healthcare workforce [24], but no focussed analysis has been conducted on the pharmacy workforce—the third largest—in Indonesia. It is imperative to conduct an analysis of pharmacy workforce capacity to identify workforce gaps for a better workforce planning strategy. The pharmacy workforce in Indonesia has diverse scopes of practice, including community; community health centres; hospitals; industry in production, quality control and quality assurance; Wholesaling or distributors to manage pharmaceutical products supply chain; and government sectors such as the National Agency of Drug and Food Control, the Ministry of Health and in the Indonesian national health insurance organisation [19]. The pharmacy workforce described in this paper was pharmacists and pharmacy technicians.

## Methods

### Data sources and data collection

Five data sources were accessed between March 2019 and December 2020 to retrieve the data used in this analysis (see Table 1). The annual headcount of registered pharmacists included graduation year and the higher education institutions (HEI)s were obtained with permission from the National Pharmacy Committee database. Pre-service capacity measures used the number of higher education institutions (HEIs) providing initial education and training, the year of HEI programme establishment, and the national number of pharmacy students per annum; these data were obtained online from the Ministry of Education website [26]. The number of eligible or potential students in the local youth population who would be able to access higher education (i.e. accessibility of pharmacy initial education and training programmes) was obtained from the Ministry of Education, Culture, Research and Technology report [27, 28]. The number of pharmacists and pharmacy assistants for each province was obtained from the Ministry of Health website [29]. Finally, a dataset from the Indonesian Pharmacists Association (IAI) was requested to obtain pharmacists’ numbers across sectors of practice, gender and age.

**Table 1:** Data sources

Data sources	Key variables	Data retrieval
National Pharmacy Committee database*	Date of birth (converted to age), gender, year of graduation, Higher Education Institutions (HEIs), license validity	28 May 2019
Ministry of Education website [26]	University, pharmacy programmes establishment date, average pharmacy students number in bachelor programmes (cohort size) in a year	20 April 2020
Ministry of Education, Culture, Research and Technology report [27, 28]	Number of general and vocational senior secondary school pupils per province	15 December 2020
Ministry of Health Website** [29]	Number of pharmacy workforce (pharmacists and pharmacy technicians) in 2019	21 April 2020, data for 31 December 2019
Indonesian Pharmacists Association (IAI) database***	Practice sector, date of birth (converted to age), gender	23 October 2021

^*^Total pharmacists: 77 191; two cases on the ‘year of graduation’ variable were missing

^**^The pharmacy workforce number included in this dataset consisted of the sum of pharmacists and pharmacy technicians who practise in the healthcare facilities (public and some private facilities). There were no trend data available for the number of pharmacists (only) in each province

^***^Total pharmacists: 78 914. There were (1) 54 331 data on the sector of practice; (2) 78 514 data on age; and (3) 78 914 data on gender

### Data analysis

All data obtained were cleaned, crosschecked and analysed using SPSS version 26. Descriptive and comparative analysis was conducted to describe the distribution of pharmacists across age, gender and sector of practice. A pharmacy workforce population pyramid was constructed to visualise and contrast the distribution of male and female pharmacist proportions between 1994 and 2019.

To analyse the workforce supply trend, the rate of change in the newly qualified/registered pharmacists is visualised over time. The supply-side function trends include a secondary axis to further compare and contrast the supply-side rate of change with the number of entry programmes in university, together with the averaged supply-side per annum to indicate growth in institutional capacity.

Correlational analysis was conducted to examine the relationships between the density of the pharmacy workforce per 10 000 population and access to university pharmacy entry programmes per 1000 potential high school students. The density of the pharmacy workforce was calculated using the following equation:

$${\text{Pharmacy}}\;{\text{workforce}}\;{\text{per}}\;10\,000\;{\text{population}} = \frac{{{\text{Number}}\;{\text{of}}\;{\text{pharmacy}}\;{\text{workforce}} \times 10\,000}}{{{\text{Population}}}}.$$

Access to entry-level pharmacy programmes was calculated using the following equation:

$${\text{Access}}\;{\text{to}}\;{\text{pharmacy}}\;{\text{programmes}} = \frac{{{\text{Capacity}}\;{\text{of}}\;{\text{pharmacy}}\;{\text{programmes}} \times 1000}}{{{\text{Potential}}\;{\text{high}}\;{\text{school}}\;{\text{students}}}}.$$

Focusing on female workforce proportion, a time-series analysis with curve estimation was conducted to estimate female workforce proportion trends and provide estimated forecasting for 2030. The time-series analysis is a specific way to analyse a collection of data points over an interval of time to forecast or predict the patterns based on these data points. When doing a time-series analysis, data points are collected at regular intervals over a predetermined time period rather than random sampling. The predictive model used 2030 as a specific year in the context of WHO’s strategic global workforce planning expectations for 2030, where there were estimated global health workforce shortages (mainly affecting low and middle-income countries). The regression projection follows the following equation [30]:

$${Y}_{t}= {b}_{0}+\left({b}_{1}\times t\right),$$

where *Y* = proportion of female pharmacists at time *t*; *b*_0_ = intercept at the vertical axis (*y* = axis); *b*_1_ = slope coefficient (or trend coefficient), and *t* = time (in years).

Measures of association were used to explore relationships between gender and sectors of practice. Analysis of variance was conducted to identify demographic differences across the sector of practice. Probability threshold was set at *p* < 0.05.

## Results

### Workforce capacity trends

**Fig. 1:**
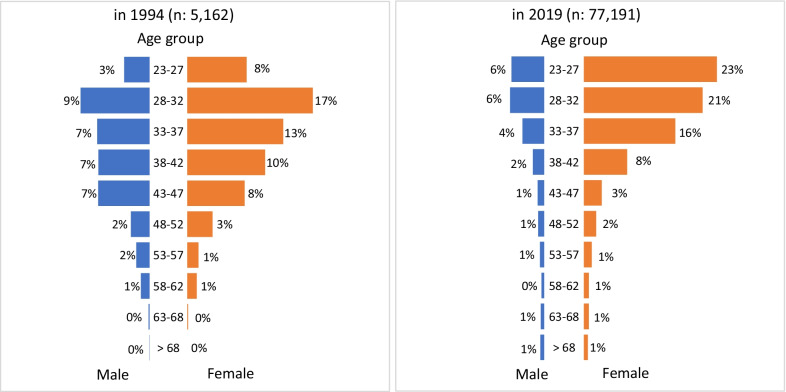
Pharmacists pyramid in 1994 (**a**) and 2019 (**b**)

Data from the National Pharmacy Committee database in 2019 showed 77 191 registered pharmacists in Indonesia (2.85 pharmacists per 10 000 population). Figure 1 shows the workforce population pyramids in 1994 (Fig. 1a) and May 2019 (Fig. 1b).

Comparing Fig. 1a and b, there has been a marked increase in graduates entering the workforce over 25 years and an increasing proportion of younger pharmacists (for example, aged 23 to 32 years) of around 19%. The workforce pyramid in 1994 (Fig. 1a) shows a ‘beehive’ structure, with the majority of the workforce aged between 28 and 47. Over 25 years, workforce growth has resulted from the increased graduate supply, as illustrated by the widening pyramid base of 2019 (Fig. 1b). Figure 1b also clearly indicates a continued growth trend in the proportion of female participation in the workforce, particularly in the younger age groups.

### Workforce supply trend

**Fig. 2:**
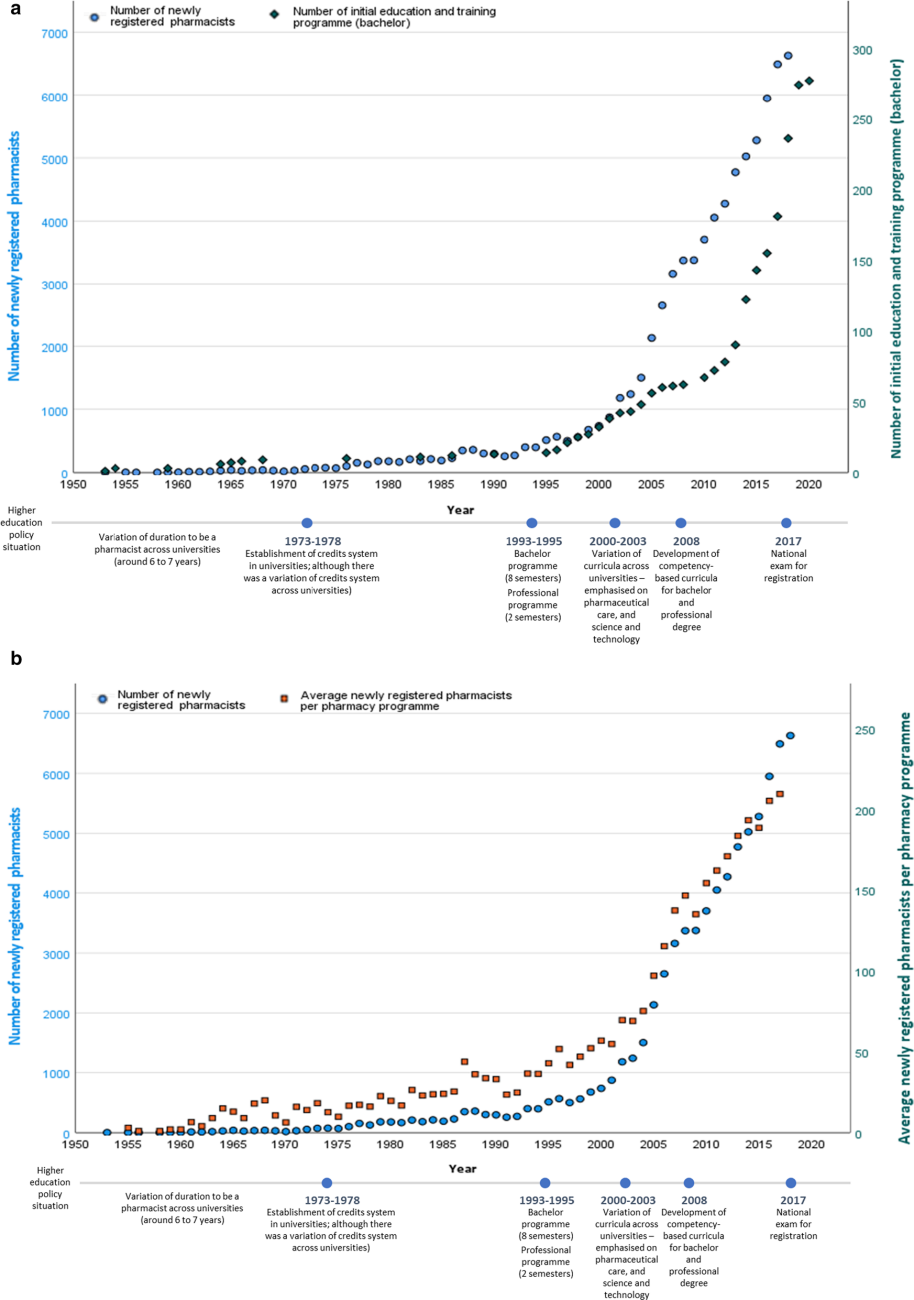
**a** Rate of change in newly registered pharmacists (left axis), the professional programme and bachelor programme number (right axis). **b** Average of newly registered pharmacists entering the workforce per annum

Figure 2a shows the rate of change in the supply of newly registered pharmacists entering the workforce from 1950 onwards (left axis—blue circle coordinates). The relationship between this supply rate change with increased access to initial education and training for undergraduates is shown in the right-hand axis of Fig. 2a (availability of university bachelor programme—green diamond coordinates). Accompanying this increased access to initial education and training programmes has been an increase in institutional capacity (student cohort size). Figure 2b shows the annual number of newly graduated pharmacists per HEI from 1955 onwards (right axis—orange square).

From 1955 to 1995, the absolute workforce trend was relatively low, with an average year-over-year growth rate of circa 23%. In 1995, several universities changed their curricula, in which the bachelor programme became an 8-semester (4 years) programme followed by a 2-semester (1 year) professional degree programme [31–33]. This curricula modification increased opportunities to open new pharmacy programmes, whereby the duration of the pharmacy programme was shorter than in earlier years. In 2000, variations of curricula across universities were reported, including a relaxation of potential student admission requirements, which led to an increasing number of student admission to each pharmacy programme. This resulted in an exponential growth of initial education and training access, which contributed to the substantial increase in the supply of newly registered pharmacists entering the national workforce. There is a general indication of the whole education market where there were increases in faculty graduation rates and capacity across the higher education institutions (HEIs) sector; both the number of pharmacy programmes and the student entry cohort sizes (measured as average graduates per pharmacy programme per year).

**Fig. 3:**
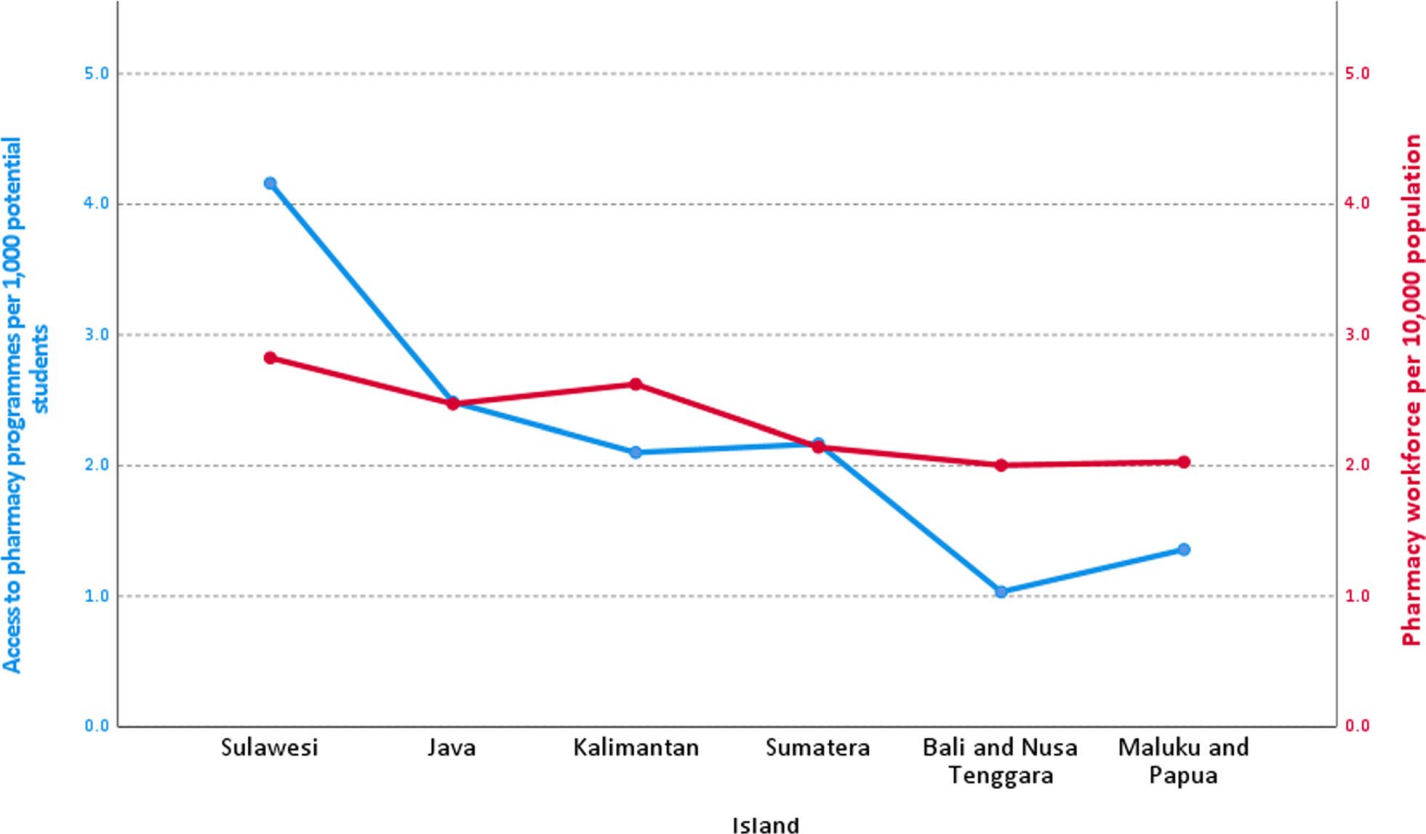
Access to pharmacy programmes per 1000 potential students and density of pharmacy workforce per 10 000 population across islands

Figure 3 compares access to pharmacy programmes for potential students and access to the pharmacy workforce for the youth population across the Indonesian islands. Compared to Sulawesi and Java islands, there was lower access to pharmacy programmes for high school students in Bali and Nusa Tenggara and with Maluku and Papua (blue line). There was also a variation in the population access to the pharmacy workforce across islands (red line), in which Sulawesi island had the highest density and Bali and Nusa Tenggara with the lowest. Correlation analysis found a strong positive correlation between the density of the pharmacy workforce per 10 000 population and access to pharmacy programmes per 1000 high school students (rho = 0.829, *p* = 0.042).

### Workforce supply trend across gender

**Fig. 4:**
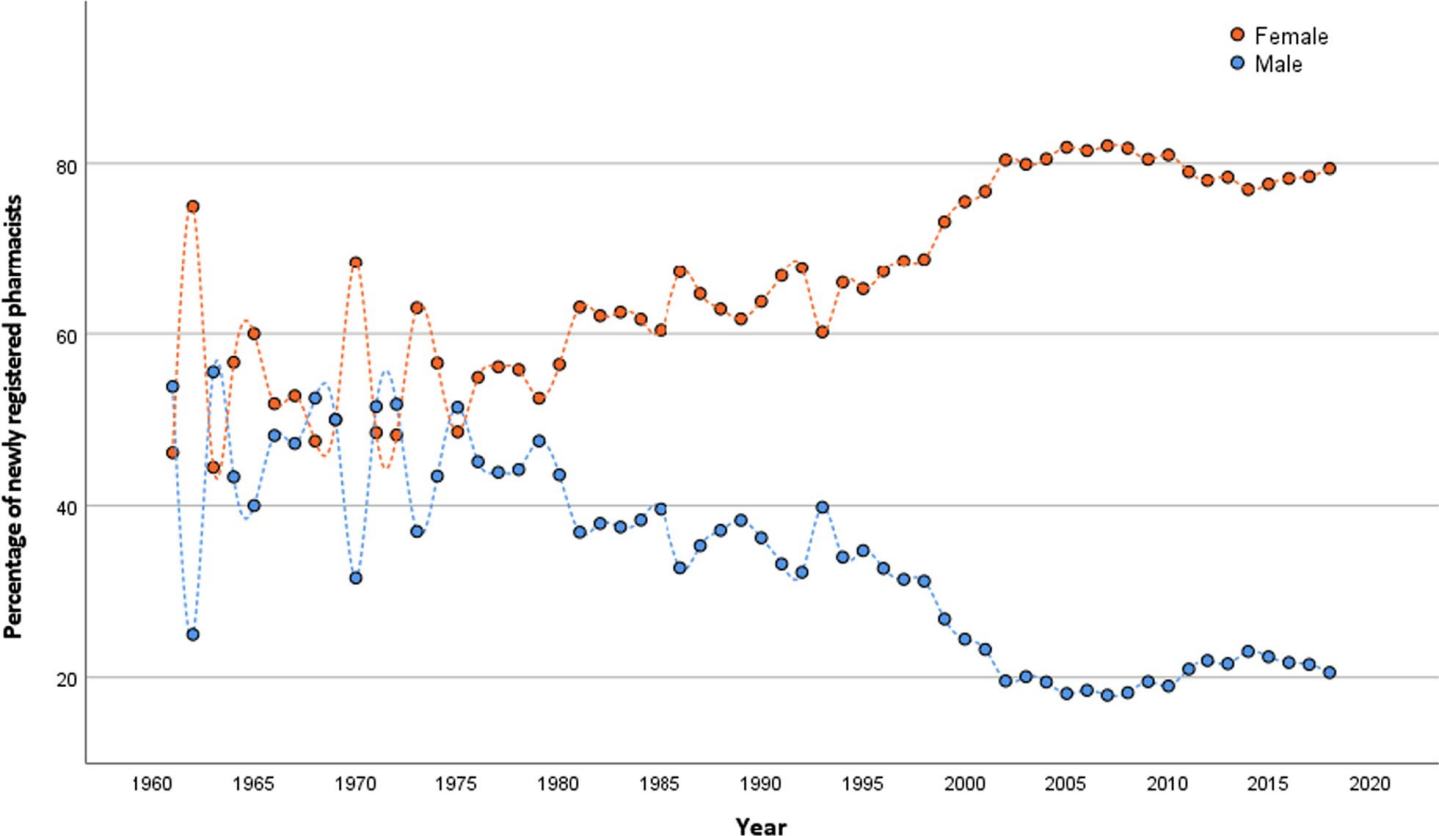
Trends in newly registered pharmacists' gender since 1955

The workforce pyramids in Fig. 1 show asymmetric proportions of males and females with an increasing trend for higher proportions of female participation. Figure 4 shows the trends of newly registered pharmacists by gender. This finding reflects the increasing proportion of females accessing higher education.

### Female pharmacists in the workforce

In the overall dataset for 2019, 78% of pharmacists in Indonesia were female. Only 50% (29 989 out of 59 943) of female pharmacists renewed their license, compared to almost 77% (13 196 out of 17 248) of male pharmacists. Based on these data trends, the regression projections for the proportion of female pharmacists follow a linear curve with the following model:

$${\text{Proportion of female pharmacists}} = 0.{51}*{\text{year}} - {949}.{5}\left( {R^{2} = 0.905, \, p < 0.0001} \right).$$

Assuming no change in linearity, or other influential variables, it is estimated that by 2030 women will represent around 86% of pharmacists in Indonesia, continuous growth of 8.2% from the most recent data.

### Pharmacist distribution across the sector of practice, age and gender

From available data on the gender and practice sector, 70.8% of pharmacists in Indonesia work in a primary healthcare sector (for example, community settings). There are significant associations between gender and the sector of practice (*χ*^2^ = 429.37, *df*5, *p* < 0.0001). More female pharmacists worked in hospital and primary healthcare (providing direct services to patients), with more male pharmacists working in the industry and Wholesaling sector.

**Fig. 5:**
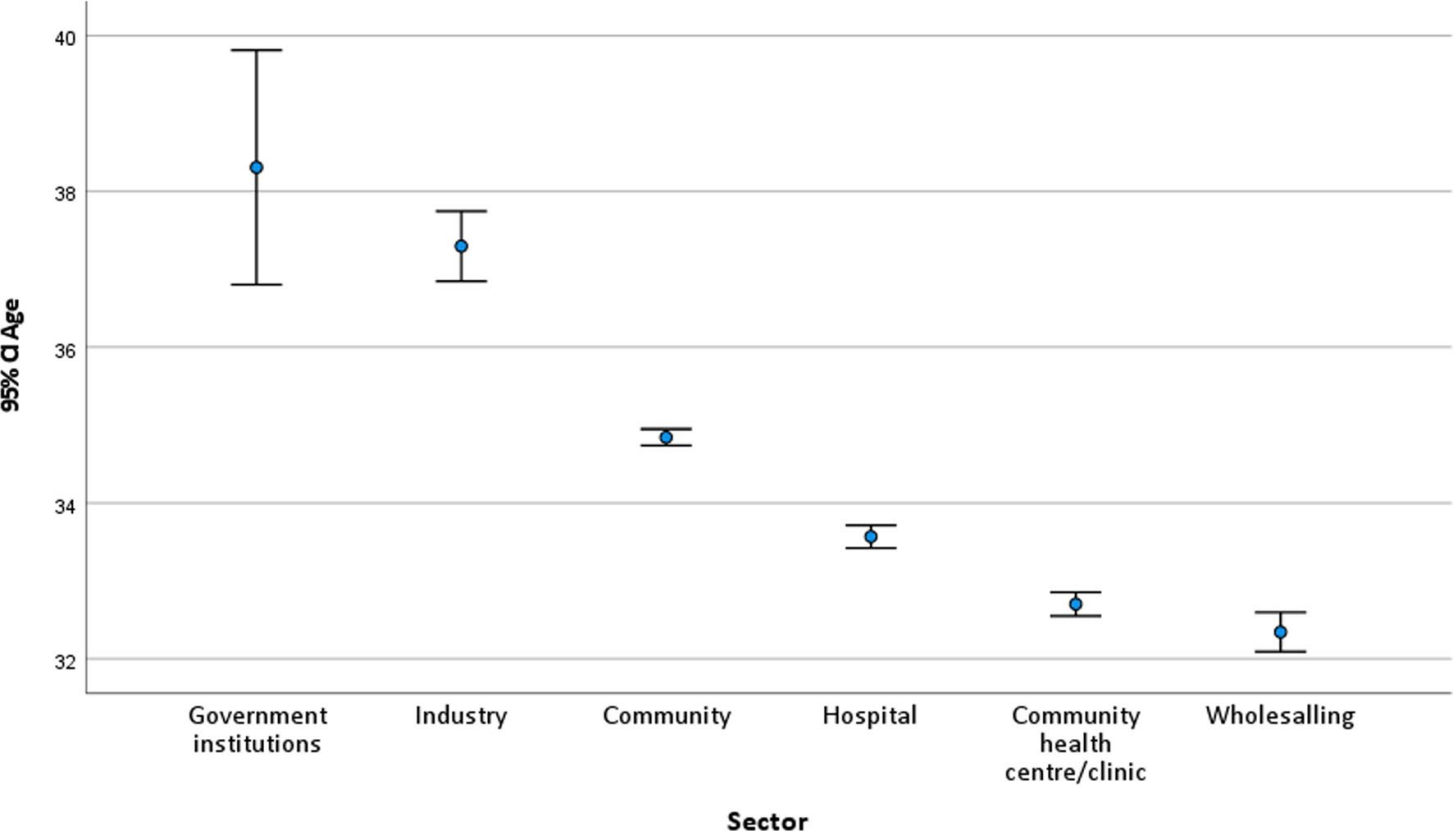
Pharmacist age distribution across the sector of practice

There was a significant age differential between the sector of practice (*F*_(5,54,324)_ = 180.145, *p* < 0.0001), with the mean age between practice sectors ranging from 32 to 38 (Fig. 5). Pharmacists working in government institutions and industry tended to be older than pharmacists working in the patient-facing role sector—community, hospital and community health centre. Pharmacists working in the Wholesaling sector were the youngest. This may indicate the attraction of this practice sector for new registrants.

## Discussion

Evidence from this study indicated an increasing trend in the number of pharmacists in Indonesia. Although there was an increasing trend, the density of pharmacists is substantially low (2.85 pharmacists per 10 000 population) compared to the global arithmetic mean in 2016 (7.36 pharmacists per 10 000 population) [12]. This may indicate sub-optimal access of the population to pharmaceutical care services in Indonesia (principally in primary healthcare environments). In addition, the pharmacists’ capacity size obtained from this study might not represent the active workforce who provide pharmaceutical care to the population—working in the patient-care setting. This study utilised data on the registration number of pharmacists, in which the data cover various sectors of practice that pharmacists can work, such as in industry, Wholesaling, government and regulatory sectors, in addition to working in patient-care settings. Further study in identifying the active pharmacy workforce and analysing the attrition pattern is needed to understand a national picture of pharmaceutical access to the population.

There was an increasing trend in the availability of higher education pharmacy initial education and training programmes number and student capacity number per programme. This indicated not only the growing market and opportunities for the pharmacy education sector, but also an effort to increase the pharmacists’ number in Indonesia. This could have resulted from the strategies by the Ministry of Health of Indonesia to strengthen HRH by improving HRH production, including the pharmacy workforce [23, 24, 34]. With an increasing supply trend resulting in an increased number of registered pharmacists, this might suggest the gap between supply and demand is decreasing. However, not all registered pharmacists will be an active workforce who provide pharmaceutical care to the population. It is also important to investigate key inflows and outflows from the workforce to gain a comprehensive analysis of the pharmacy workforce. The key inflows are: registration of new graduates, immigration and return to work following inactivity; the key outflows are retirement, emigration, career break and death in service [35]. In this study, the researcher only analysed the registration of new graduates. There was no information on immigration, return to work, emigration, career break, retirement or death specific to the pharmacy workforce. It is important to have these data to understand supply and productivity. Further research is also needed to understand how the supply has met the demand.

This study found that there was a variety of access to pharmacy programmes across islands. The inequality in access could contribute to the inequality of distribution of pharmacists. This study found a strong positive correlation between access to pharmacy programmes and access to the pharmacy workforce, indicating a possibility that the location of pharmacy schools might influence where students/graduates were based and looking for jobs. In Indonesia, the University in Indonesia in Depok and Padjajaran University in Jatinangor, West Java, are examples of successful higher-education facilities that draw migrants away from urban regions [36]. This study supports evidence from previous research in other countries, which found that rurally based faculties and healthcare schools provided a majority of graduates to the local rural healthcare workforce [37, 38]. Similarly, graduates from urban settings were more likely to remain and work in these urban environments [37, 38]. The WHO recommended further studies to explore the effects of locating schools and programmes outside urban areas on subsequent employment [39].

This study showed that there is an imbalance in the distribution of the pharmacy workforce across islands in Indonesia. This finding seems consistent with other research that found an uneven distribution of the mixed healthcare workforce between rural and urban areas in Indonesia [40–42]. A survey conducted in 2011 found variation across islands ranging from 23.2 to 51.5% of community health centres did not have pharmacists and pharmacy support staff to provide pharmaceutical care and medicines expertise, particularly in the Eastern Indonesia region [43]. The uneven distribution and unavailability of the pharmacy workforce illustrate that accessibility to medicines expertise, in the face of increasing use of medicines in an ageing co-morbid population, may have repercussions for universal health coverage and sub-optimal primary healthcare support. The pattern of having a higher workforce density in the capital city is also typical of other low- and lower-middle-income countries [44–46]. Reasons for this urban imbalance might be family and social relationships, working and living conditions, career opportunities and financial incentives [47]. In Indonesia, this pattern is also seen among other health professionals. An analysis in 2019 of general mixed health workforce distribution (physicians, nurses and midwives) using the Gini Index in Indonesia found that the number of physicians and nurses was concentrated in the capital of provinces or other big cities in the province [48]. Since 2015, the Ministry of Health of Indonesia has formulated *the Nusantara Sehat* programme for special recruitment to recruit health workers, including pharmacists, to work in community health centres in remote areas [49]. The evidence from this study suggests the need for more policies and strategies that would facilitate the recruitment and retention of the pharmacy workforce in rural and remote areas.

An increase in pharmacists’ graduation rate (supply pipeline) resulted in a youth bulge of pharmacists aged between 23 and 37. This situation suggests a need to develop structured post-license foundation training to better support early career pharmacists. Having structured foundation training in place will support early career pharmacists to progress toward advanced practice, and opportunities to engage with peers and preceptors are essential to ensure their readiness for practice. Opportunities for pharmacists have grown, considering the expanding role of pharmacists as medicines experts and the population health challenges, e.g., the ageing population; therefore, the existing workforce needs to scale up advanced training competencies to be more flexible and adaptable to face rising demand and healthcare challenges. Developing a programme to support pharmacists in developing their advanced practice competencies is urged. Moreover, opportunities for education and training and support to the workforce were found to be associated with job satisfaction for early career pharmacists, and job dissatisfaction was found to be linked with a higher attrition rate [50]. Therefore providing structured training and opportunities for advanced training to support early career pharmacists could have an impact on increasing workforce retention.

Like the global pharmacy workforce trends, this study found more females (77.8%) than male pharmacists in Indonesia and an increasing trend was predicted. Females in the pharmacy workforce generally have more career breaks than males and the high proportion of female pharmacists with family responsibilities, and increasingly early retirement ages, are factors that need to be considered more urgently for workforce planning [10]. In 2019, the general labour force participation rate of females aged 15–64 in Indonesia was 56%, significantly lower than 84% for males [51]. The Ministry of Manpower of Indonesia called for flexible working conditions for women to increase this participation rate [52, 53]. It is essential to develop a professional development system for female practitioners, allowing flexibility in professional development to facilitate them returning from a career break. This study also observed a lower license renewal rate for female pharmacists. This is an indication that ‘return to work’ policies need greater emphasis in Indonesia.

Another important finding from this study was that younger pharmacists tended to work in the Wholesaling sector after graduating more than in the other sectors of practice. They may be more attracted to work in the Wholesaling sector, which is possibly related to higher remuneration for early career pharmacists [54–56]. This may also be because of a recent expansion of industry markets in Indonesia, which might attract more recent graduates to take up more opportunities. More older pharmacists worked in government institutions and industry settings, which may reflect a more static job market and more opportunities in these sectors, tending to retain existing employees (and hence the strata getting older because they stay in the job for longer).

## Limitation

One of the limitations of this study was related to data integrity. This study used several sources to extract and analyse data, with each source having implicit limitations. An integrated system to collect all pharmacist data in Indonesia is imperative to improve the dataset’s accuracy and analysis. The Indonesian Pharmacists Association is currently developing an intelligence system, the Pharmacy Informational System (*Sistem Informasi Apoteker*-SIAp). This is also in line with a strategy by the Ministry of Health in HRH, which is to strengthen health workforce information systems and build a data-driven culture to address HRH challenges [22–25].

To develop a more in-depth analysis of pharmacy workforce capacity, some following information is needed. First, there was no information found about the proportion of inactive pharmacists. It is important to identify the actual number of pharmacists who provide services to understand pharmaceutical care availability in Indonesian society. Second, information on the working time (part-time or full-time) is important to identify attrition patterns. There was no information on this in the current dataset. Third, information on what had happened to pharmacists who did not re-apply for their licence was not available. This finding will help to give a better picture of what happens in the workforce. Fourth, there was a limitation on the sector of practice, particularly related to changing employment to a field outside pharmacy, which could not be identified. Having this information will help in suggesting a policy on the utilisation of existing pharmacists. Fifth, the availability of data on occupation and earnings may enable a more detailed analysis of gender equality in the health workforce. Expanding these data is essential to develop evidence informing workforce policies.

## Conclusions

This study is the first in-depth study to analyse the pharmacy workforce data in Indonesia. This study provided a comprehensive overview of the pharmacy workforce which could be used as a template for further longitudinal analysis by the professional leadership body in Indonesia. The data showed there were access imbalances of the population to pharmacy workforce by geographical region, suggesting policies and strategies to facilitate recruitment of pharmacy workforce, particularly in rural areas. The imbalance of female-to-male participation in the pharmacy workforce also has implications for workforce planning and providing greater opportunities for retaining females within the workforce. Moreover, this study provided evidence on the analysis of the pharmacy workforce in the South East Asia region, which could offer guidance to the policymakers at the local level and at the regional and global levels. To build a data-driven culture in addressing health workforce challenges, it is imperative to strengthen the pharmacy intelligence system.

## Data Availability

The data that support the findings of this study are available from the Indonesian Pharmacist Association, but restrictions apply to the availability of these data, which were used under license for the current study, and so are not publicly available. Data are, however, available from the authors upon reasonable request and with permission of the Indonesian Pharmacist Association.

## Abbreviations

DG: Development goals
FIP: International Pharmaceutical Federation
HEI: Higher Education Institution
HRH: Human Resources for Health
IAI: Indonesian Pharmacists Association
NCDs: Non-communicable diseases
SIAp: Pharmacy Informational System
SPSS: Statistical Package for the Social Sciences
WHO: World Health Organization
